# Advancing Non-Invasive Colorectal Cancer Screening: Exploring the Potential of Monoclonal Antibody L2A5

**DOI:** 10.3390/ijms26073070

**Published:** 2025-03-27

**Authors:** Renato Caldevilla, Mariana Eiras, Daniela A. R. Santos, João Almeida, Beatriz Oliveira, Susana Loureiro, Janine Soares, Miguel Gonzalez-Santos, Nuno Ramos, Paula A. Videira, Lúcio Lara Santos, Mário Dinis-Ribeiro, Luís Lima

**Affiliations:** 1 Experimental Pathology and Therapeutics Group, Research Centre of IPO Porto (CI-IPOP), RISE@CI-IPOP (Health Research Network), Portuguese Oncology Institute of Porto (IPO Porto), Porto Comprehensive Cancer Centre Raquel Seruca (Porto.CCC Raquel Seruca), 4200-072 Porto, Portugal; i40125@ipoporto.min-saude.pt (R.C.); mariana.eiras@ipoporto.min-saude.pt (M.E.); daniela.r.santos@ipoporto.min-saude.pt (D.A.R.S.); joao.almeida@i3s.up.pt (J.A.); bia_faria2@hotmail.com (B.O.); i40111@ipoporto.min-saude.pt (S.L.); eliana.paiva.soares@ipoporto.min-saude.pt (J.S.); m.gonzalezsantos@gmail.com (M.G.-S.); lucio.santos@ipoporto.min-saude.pt (L.L.S.); 2 Institute of Biomedical Sciences Abel Salazar (ICBAS), University of Porto, 4050-313 Porto, Portugal; 3 Faculty of Medicine (FMUP), University of Porto, 4200-072 Porto, Portugal; mario.ribeiro@ipoporto.min-saude.pt; 4 School of Health (E2S), Polytechnic Institute of Porto, Rua Dr. António Bernardino de Almeida, 400, 4200-072 Porto, Portugal; 5 UCIBIO—Applied Molecular Biosciences Unit, Department of Life Sciences, NOVA School of Science and Technology, Universidade NOVA de Lisboa, 2829-516 Caparica, Portugal; npregoramos@sapo.pt (N.R.); p.videira@fct.unl.pt (P.A.V.); 6 Associate Laboratory i4HB—Institute for Health and Bioeconomy, NOVA School of Science and Technology, Universidade NOVA de Lisboa, 2829-516 Caparica, Portugal; 7 CDG & Allies-Professionals and Patient Associations International Network, 2819-516 Caparica, Portugal; 8 FF-I3ID, University Fernando Pessoa, 4249-004 Porto, Portugal; 9 GlycoMatters Biotech, 4500-162 Espinho, Portugal; 10 Department of Surgical Oncology, Portuguese Oncology Institute of Porto (IPO-Porto), 4200-072 Porto, Portugal; 11 Precancerous Lesions and Early Cancer Management Group, Research Centre of IPO Porto (CI-IPOP), Rise@CI-IPOP (Health Research Group), Portuguese Institute of Oncology of Porto (IPO Porto), Porto Comprehensive Cancer Centre Raquel Seruca (Porto.CCC Raquel Seruca), 4200-072 Porto, Portugal; 12 Department of Gastroenterology, Portuguese Oncology Institute of Porto, 4200-072 Porto, Portugal

**Keywords:** colorectal cancer, early screening, L2A5 antibody, precancerous lesions, sialyl-Tn, stool samples

## Abstract

Early detection of colorectal cancer (CRC) significantly improves overall prognosis and increases 5-year survival rates up to 90%. Current non-invasive screening methods for CRC, such as the Faecal Immunohistochemical Test (FIT), have some drawbacks, namely, low sensitivity and a high false-positive rate. The Sialyl-Tn (STn) antigen, frequently expressed in pre-malignant lesions and adenocarcinomas, has been shown to be detected by the novel monoclonal antibody L2A5. In this study, we explored the potential of L2A5 as a non-invasive CRC screening method in an attempt to overcome current limitations. The subjects were categorised into four groups based on colonoscopy findings: no lesion (NL), low-grade dysplasia (LGD), high-grade dysplasia (HGD), and colorectal cancer (CRC). Slot blot analysis using the L2A5 antibody was performed on stool samples from 95 colonoscopy patients. Our findings showed a differential STn expression between the different clinical groups, evidencing excellent discrimination between NL and CRC (AUC, 0.8252; 95% CI: 0.6983–0.9521; sensitivity, 70%). Moreover, moderate discrimination between the NL+LGD and HGD+CRC groups was discerned (AUC, 0.7766; 95% CI: 0.6792–0.8740; sensitivity, 58%). These findings support the application of L2A5 as a tool for detecting STn, allowing for the identification of advanced lesions in non-invasive CRC screening.

## 1. Introduction

Colorectal cancer (CRC) is the second leading cause of cancer-related death worldwide and the third most common type of cancer [1]. The slow progression of the disease over 10 years allows for the opportunity for early cancer screening and pre-malignant lesions resection [2].

One of the primary CRC screening methods involves non-invasive stool-based tests designed to detect occult blood in faeces, which is followed by a confirmatory colonoscopy [3]. The most broadly implemented non-invasive test is the Faecal Immunochemical Test (FIT) due to its high sensitivity (70–80%) and specificity (90–95%) in tumour detection [4,5]. However, considering the importance of high sensitivity in screening methods, it is important to highlight the main downside of FIT, namely, its 30–40% sensitivity range in detecting precancerous lesions, leading to missed detection of CRC cases [3,4,6]. FIT’s high false-positive rates also lead to unnecessary colonoscopies and an increased healthcare burden. Colonoscopy remains the gold standard for CRC screening—as it enables direct visualisation of the colon and rectum while simultaneously facilitating the detection and removal of lesions—with a sensitivity of over 90% for CRC [7]. However, this invasive approach includes some major drawbacks, namely, intrinsic procedure complications, such as bowel perforation or bleeding, extensive bowel preparation prerequisites, and sedation or anaesthesia requirements [8,9]. Consequently, it negatively impacts population adherence rates [10].

The lasting hurdle in CRC screening for early tumour detection is the absence of non-invasive tools with high sensitivity and specificity that outperform FIT and colonoscopy without additional risks and costs, which could significantly improve compliance rates.

Over the years, a diverse range of potential biomarkers have been extensively studied as possible non-invasive approaches for CRC screening [11]. Aberrant glycosylation is a key event in CRC progression, regulating tumour progression, invasion, and metastasis [12,13]. While the detection of truncated *O*-glycans in normal epithelia is rare, it is frequently observed in cancer, namely, in colorectal (80%), breast (57%), and pancreatic (56%) cancer [12,14]. A previous study by our team identified abnormal glycan signatures, namely, of the Tn antigen, in the stool of individuals with aggressive lesions compared to healthy tissues, paving the way for glycosylation alterations as a tool for non-invasive CRC detection [15]. This intricate interplay of glycosylation dysregulation in cancer highlights the potential for a targeted approach. Sialyl-Tn (STn) is expressed in approximately 80% of human carcinomas and is known to drive cancer progression and aggressiveness [14,16,17]. This glycan is expressed in many types of digestive cancers, including oesophagus carcinoma, gastric cancer, and CRC [18]. Studies in CRC have demonstrated the clear cancer-associated nature of STn and its potential application in therapeutics [14,18,19,20,21]. A monoclonal antibody L2A5, with the ability to target core STn and α2-6- linked sialyl core-1 probes, has already been developed [22]. This antibody is characterised by a unique binding specificity and high sensitivity, exhibiting reactivity from low-grade to invasive fronts of CRC tumour tissues, outperforming commonly used anti-STn antibodies in binding affinity, as confirmed by a wide range of assays. Given its distinct affinity profile, it is proposed as a potential diagnostic tool for CRC [22]. Recognising the potential of STn as a candidate biomarker and aiming to address the limitations in well-established CRC non-invasive screening methods, this study is the first to evaluate the L2A5-STn duo as a screening tool in stool samples.

## 2. Results

### 2.1. Antibody Binding Optimisation Using BSM

**Figure 1:**
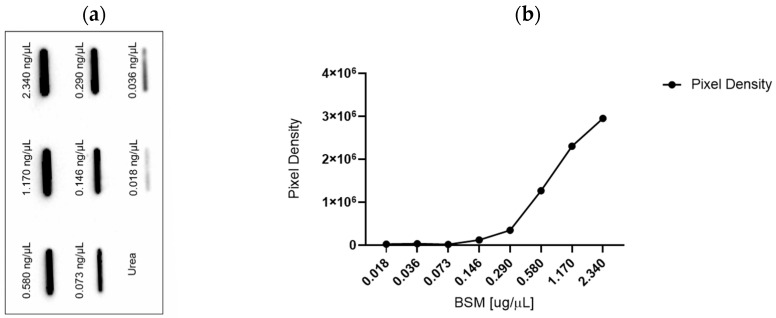
The antigen L2A5 demonstrated high sensitivity and strong affinity for low protein concentrations. (**a**) A slot blot analysis of a series of progressively lower protein concentrations was performed, with positive antigen-binding signals detected at all levels. A weaker signal is observed with a lower protein input. (**b**) A significant correlation was observed between protein concentration and band signal intensity, measured as pixel density (*p* = 0.0001).

The sensitivity of the L2A5 antibody’s binding capacity was initially assessed using a baseline range of protein inputs. Bovine Submaxillary Mucin (BSM) is enriched for STn expression, whereas urea lacks the expression of this glycan. Thus, they were used as positive and negative controls, respectively. Binding affinity was successfully observed across several concentration inputs ranging from 0.018 to 2.34 ng/µL, demonstrating a correlation between protein concentration and signal intensity (Figure 1).

### 2.2. L2A5 Screening Potential

**Figure 2:**
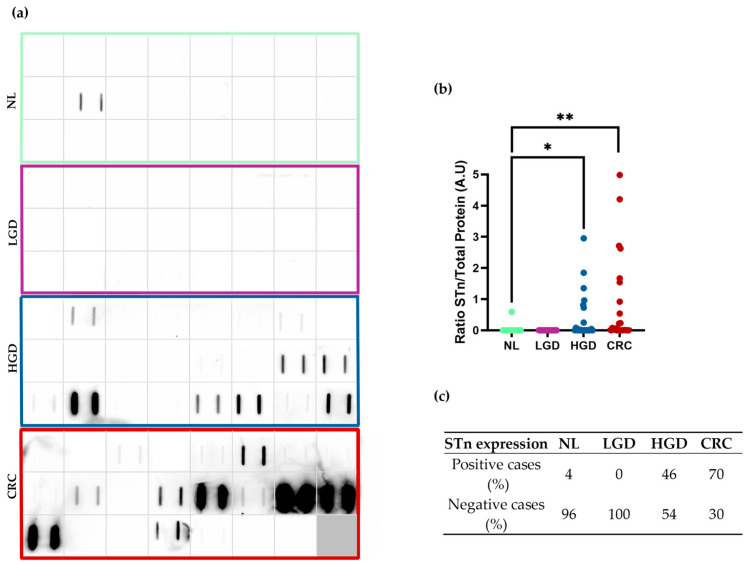
Assessment of L2A5 screening potential in stool samples. (**a**) STn expression in 95 stool samples from different clinical groups: NL, LGD, HGD and CRC. Slot-blot of the stool samples was performed in duplicate. Squares represent each patient, and two biological replicates were used. STn-positive samples are featured in black. NL, LGD, HGD and CRC are outlined in green, purple, blue and red, respectively. (**b**) STn stool expression levels among the different evaluated groups. Higher levels of STn were exhibited in the HGD and CRC groups, when compared to the NL group, with statistical significance for both (* HGD *p*-value = 0.0261; and ** CRC *p*-value = 0.0070). (**c**) Distribution of STn-positive cases among the analysed groups. Abbreviations: NL—No Lesion; LGD—Low-Grade Dysplasia; HGD—High-Grade Dysplasia; CRC—Colorectal Cancer.

To evaluate the potential of monoclonal antibody L2A5 to detect STn in stool, its expression was analysed in 95 samples by Slot-Blot analysis (Figure 2a). The correlation between demographic and clinicopathological variables and STn expression was assessed (Supplementary Table S1). These findings suggest that both polyp multiplicity (*p* = 0.017) and increased size (*p* = 0.013) are associated with the detection of STn antigen. No further correlations were established between STn expression and other variables.

According to the morphological and histological characteristics of the lesions, samples were categorised into four clinical groups: No Lesion (NL), Low-Grade Dysplasia (LGD), High-Grade Dysplasia (HGD), and CRC.

The results indicate that STn expression levels vary among the different clinical groups. The antigen expression was significantly higher in samples from the HGD group compared to the NL group (0.3536 ± 0.1538) (Figure 2b). Furthermore, a difference with the NL group was also observed in CRC samples (0.8379 ± 0.2964) (Figure 2b).

Sialyl-Tn was expressed in only one sample from the NL group, while no samples from the LGD group tested positive for the presence of STn. Nevertheless, 46% and 70% of the HGD and CRC cases, respectively, were positive for STn expression. These findings suggest that the detection of STn antigen is limited or less prevalent in the NL and LGD groups.

Subsequently, to further evaluate the potential of L2A5 in identifying advanced lesions (HGD and CRC), the four clinical groups were combined into two major groups: the non-relevant findings (NRF) and HGD+CRC. The NRF group—including the samples from the NL and LGD groups—represents cases with a lower risk of developing into cancer. Conversely, the HGD+CRC group includes individuals with high-grade dysplasia adenomas or diagnosed with colorectal cancer.

**Figure 3:**
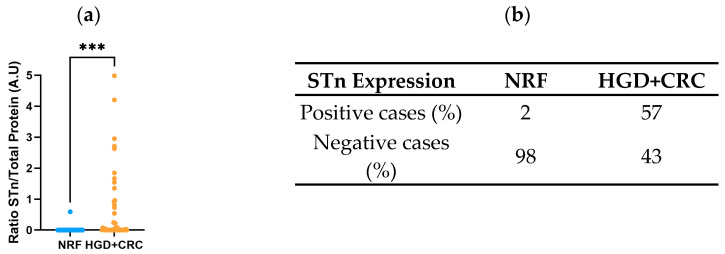
Screening potential of L2A5 antibody in advanced lesions. (**a**) Graphical representation of STn antigen levels in the combined groups based on their aggressiveness. STn expression is mostly expressed in advanced lesions and its levels are significantly higher in this group (HGD+CRC) in comparison with the one containing healthy patients or low-grade dysplasia lesions (NRF); *** *p* = 0.0005. (**b**) Distribution of STn presence in NRF and HGD+CRC groups. Abbreviations: NRF—Non-Relevant Findings; HGD—High-Grade Dysplasia; CRC—Colorectal Cancer.

Using L2A5, we found that STn expression was significantly higher in the HGD+CRC group compared to the NRF group (0.6030 ± 0.1674, *p* = 0.0005) (Figure 3a). These results suggest the potential of STn expression to identify and differentiate individuals with advanced colorectal lesions, such as HGD and cancer, from NRF lesions (NL+LGD). Additionally, the distribution of STn among these two groups further supports these findings since only 2% of NRF cases are STn-positive versus 57% in the HGD+CRC group (Figure 3b).

### 2.3. Clinical Value of STn in Cancerous Lesions Detection

An ROC curve analysis was performed to assess the clinical value of STn in detecting cancerous lesions (Supplementary Figure S1). Based on the AUC values, poor discrimination between LGD and NL groups was verified (AUC, 0.5208; 95% CI: 0.3560–0.6857). Nonetheless, moderate discrimination power was observed between the HGD and NL groups (AUC, 0.7092; 95% CI: 0.5597–0.8587), with a sensitivity of 46% and specificity of 96% for HGD lesion detection. Excellent discrimination power was observed between the CRC and NL groups (AUC, 0.8252; 95% CI: 0.6983–0.9521), with good sensitivity and specificity (70% and 96%, respectively). Likewise, moderate discrimination between the NRF (NL+LGD) and HGD+CRC groups was discerned (AUC, 0.7766; 95% CI: 0.6792–0.8740), with a sensitivity of 58% and specificity of 98% (Supplementary Table S2).

## 3. Discussion

The primary obstacle in CRC screening remains the development of a non-invasive diagnostic tool that surpasses FIT’s sensitivity and specificity, namely, for detecting advanced adenomas. Moreover, given the increasing focus on biomarker-based approaches over the past few years, we addressed the potential of glycosylation alterations, specifically STn, as a screening tool for CRC.

CRC screening burden is largely attributed to the high number of individuals undergoing unnecessary colonoscopy procedures, including those with no lesions or low-grade dysplasia adenomas [23,24]. Reducing this burden while enhancing the accuracy of the identification of individuals who urgently need medical intervention is thus imperative. Furthermore, an effective screening approach must prioritise the detection of individuals with high-grade dysplasia lesions, ensuring adequate clinical management and follow-up, as these carry an increased risk of progression to advanced disease [24].

As current CRC screening tools employ stool as the main biofluid, we evaluated the applicability of L2A5 monoclonal antibody as a tool for CRC screening in stool samples. To our knowledge, this is the first study to assess STn expression in this sample type.

In the context of CRC screening, it is crucial that biomarkers exhibit high sensitivity that accurately discriminates individuals with advanced lesions. In this study, STn expression levels allowed for the discrimination between NL and both HGD and CRC with high specificity (96%) and sensitivities of 46% and 70%, respectively. Notably, our findings indicate an increase of approximately 15 percentage points in advanced adenoma detection compared to FIT, while maintaining CRC detection sensitivity. The clinical value of our findings suggests that STn has the potential to moderately discriminate individuals with HGD or CRC from the remaining population.

Moreover, accurately detecting individuals with LGD lesions could constitute a hallmark for the reduction in colonoscopy burden as this phenotype is associated with a lower risk of progression to advanced disease. With regular screening, LGD individuals could be monitored over time and identified if their lesions evolve into advanced adenomas. Therefore, the strategy here reported could help reduce the post-screening-test colonoscopy burden since 98% of individuals with no lesions or LGD were accurately identified—a finding that can be a particularly appealing prospect for clinicians.

Nevertheless, a more robust body of evidence and further validation of this approach to a wider sample set, including FIT negative patients, could strengthen our findings and allow for a more accurate comparison with the performance of current screening methods, such as FIT.

Here, we focused on the use of L2A5 to detect STn, using a Slot-Blot analysis—an affordable, easy-to-use, and rapid technique for protein quantification. This technique is also better fitted than other methodologies for binary (positive/negative) identification and allows for clear discrimination between the groups of interest. Nevertheless, future research should focus on quantifying STn expression levels through a quantitative antigen-based strategy, such as ELISA, which could further explore the L2A5 discriminatory potential. Moreover, Slot-Blot’s principles and workflow seem more suitable for the development of a rapid, accurate, cost-effective, and easy-to-use point-of-care device, highlighting the potential of its clinical application.

Unveiling new frontiers in non-invasive CRC screening, this study delves into the promising role of the L2A5-STn dyad in stool samples. The observed discrimination between the clinical groups, namely, HGD and CRC patients, allows for the detection of cancer-related features present in individuals with precancerous and cancerous lesions, enabling the indication for further procedures.

The application of L2A5 antibody in stool samples is an innovative approach. Given the exploratory nature of this study, the results obtained using this antibody in stool samples should be interpreted with caution. Future studies should address these limitations by expanding the study population and conducting a more thorough validation, thus providing a clearer overview of STn’s diagnostic utility in early CRC screening.

Finally, this work highlights the unmapped potential of L2A5 antibody for non-invasive CRC screening, providing a framework for addressing current screening tools’ limitations.

## 4. Materials and Methods

### 4.1. Patient Sample Set

A case–control study was conducted at the Gastroenterology Department of IPO-Porto, recruiting individuals participating in the Portuguese Colorectal Cancer screening program. Between April 2022 and July 2023, after a positive Faecal Immunochemical Test (FIT) result and prior to their scheduled colonoscopy, a total of 95 stool samples were freshly collected, homogenised, coded, and stored at −80 °C. According to the colonoscopy findings, the samples were categorised into four similarly sized groups: individuals with no lesions in the colon and rectum (NL), with at least one low-grade dysplasia lesion (LGD), with at least one high-grade dysplasia lesion (HGD), and patients diagnosed with colorectal cancer (CRC). The median age of individuals was 62 years (min–max: 49–89 years), and the male percentage was approximately 60% (Table 1). This study received approval from the IPO-Porto’s Ethical and Data Protection Committee (approval number CES 22/022, dated 10 February 2022) and was conducted following international guidelines, whereby all individuals provided written informed consent.

### 4.2. Protein Extraction and Precipitation

Total protein extraction was performed from an input of 100 mg of stool sample. A solution containing RIPA buffer (50 mM Tris at pH 8, 150 mM NaCl, 1% NP40, 0.5% deoxycholate, and 0.1% SDS) and proteinase inhibitors (aprotinin, leupeptin, PMSF, NaF, NaVO_3_, Na_4_P_2_O_7_) was prepared and added to each sample. After a 20 min incubation on ice, the samples were centrifuged at 2000× *g* for 10 min at 4 °C, and the supernatant was collected and preserved. Proteins were further precipitated following methanol–chloroform incubations. Briefly, methanol (4:1), chloroform (1:1), and MiliQ Water (3:1) were added sequentially to the extracted proteins. Afterwards, the solution was centrifuged at 14,000× *g* for 1 min at 4 °C. The upper aqueous layer was collected, and methanol (400 μL) was added to the supernatant. Then, the mixture was centrifuged at 14,000× *g* for 2 min at 4 °C, the supernatant was discarded, and the resulting pellet was air-dried. Afterwards, the pellet was resuspended with urea 1M, vortexed, and sonicated for a maximum dissolution of protein in the buffer. The protein precipitation mixture was heated at 80 °C for 3 min and centrifuged at 14,000× *g* for 15 min at RT. The supernatant was collected, and protein integrity was assessed through SDS-Page. Protein quantification was determined using the DC Protein assay (Bio-Rad, Hercules, CA, USA).

### 4.3. L2A5 Blotting

Stool proteins (50 μg) were slot-blotted on a nitrocellulose membrane (AmershamTMProtranTM, Cytiva, Marlborough, MA, USA) using Hybri-Slot apparatus (21052-014; Gibco BRL, Life Technologies, Waltham, MA, USA). The membrane was blocked with Carbo-Free Blocking Solution for 2 h at RT. Proteins were blotted with the primary antibody L2A5 (mlgG1 version) (1:2500; CellmAbs) for 1 h at RT. The goat anti-mouse IgG (H+L) polyclonal antibody (1:70,000, ImmunoResearch) was used as a secondary antibody and incubated for 30 min at RT. The Amersham ECL Prime Detection Reagent was used as developing reagent. Data analysis was performed through Image Lab Software (version 6.0.1) (Bio-Rad, Hercules, CA, USA) in ChemiDoc XRS (Bio-Rad, Hercules, CA, USA).

### 4.4. Statistical Analysis

Data analysis was performed using SPSS (version 28.0, IBM, Inc., Chicago, IL, USA) and GraphPad Prism 10.0 (GraphPad Software, Inc., San Diego, CA, USA) for visualisation. The Shapiro–Wilk test assessed data normality, while the Mann–Whitney U test analysed continuous variables. Outliers were identified and *p*-values < 0.05 were considered significant. Chi-square analysis was used to compare categorical variables. The clinical value was assessed using receiver operating characteristic (ROC) curves. The optimal threshold cutoff values were determined by maximising the Youden Index (=sensitivity + specificity−1). The AUC values were classified as follows: AUC of 0.5—no discrimination; AUC 0.6–0.7—acceptable discrimination; AUC 0.7–0.8—moderate discrimination; AUC 0.8–0.9 —excellent discrimination; and AUC 0.9–1.0—outstanding discrimination [25].

## Figures and Tables

**Table 1:** Demographic and clinicopathological data associated with stool samples (n = 95).

Characteristics	No Lesion	Low-Grade Dysplasia	High-Grade Dysplasia	Colorectal Cancer
**N**	24	24	24	23
**Age** (mean, min–max)	59 (50–72)	57 (49–74)	64 (50–84)	67 (51–89)
**Sex n(%)**				
Female	10 (40)	8 (40)	11 (44)	10 (44)
Male	14 (60)	16 (60)	13 (56)	13 (56)
**Localisation n(%)**				
Right colon	-	7 (29)	6 (25)	6 (26)
Left colon	-	17 (71)	14 (71)	3 (13)
Rectum	-	0	1 (4)	14 (61)
**Number of polyps n(%)**				
<2	-	16 (67)	12 (50)	-
≥3	-	8 (33)	12 (50)	-
**Size of polyps n(%)**				
<1 mm	-	16 (67)	4 (17)	-
≥1 mm	-	8 (33)	19 (83)	-
**TNM stage n(%)**				
**Tumour (T)**				
T1	-	-	-	7 (30)
T2	-	-	-	4 (17)
T3	-	-	-	10 (44)
T4	-	-	-	2 (9)
**Lymph node metastasis (N)**				
N0	-	-	-	19 (83)
N1	-	-	-	1 (4)
N2	-	-	-	3 (13)
N3	-	-	-	0
**Metastasis (M)**				
M0	-	-	-	23 (100)
M1	-	-	-	0
**Clinical Stage n(%)**				
I	-	-	-	9 (39)
II	-	-	-	9 (39)
III	-	-	-	5 (22)
IV	-	-	-	0

## Data Availability

The data generated in this study are included in the main article or Supplementary Materials.

## Supplementary Materials

The following supporting information can be downloaded at: https://www.mdpi.com/article/10.3390/ijms26073070/s1.
