# Supplementary material for: Advancing Non-Invasive Colorectal Cancer Screening: Exploring the Potential of Monoclonal Antibody L2A5
Source: Int J Mol Sci. 2025 Mar 27;26(7):3070. doi: 10.3390/ijms26073070 (PMC11988763; doi:10.3390/ijms26073070)
Supplement: Supplementary file 1 [file ijms-26-03070-s001.zip › ijms-3488384-supplementary.pdf]

Advancing Non-Invasive Colorectal Cancer Screening: Exploring the Potential of Monoclonal Antibody L2A5

-Supporting information-

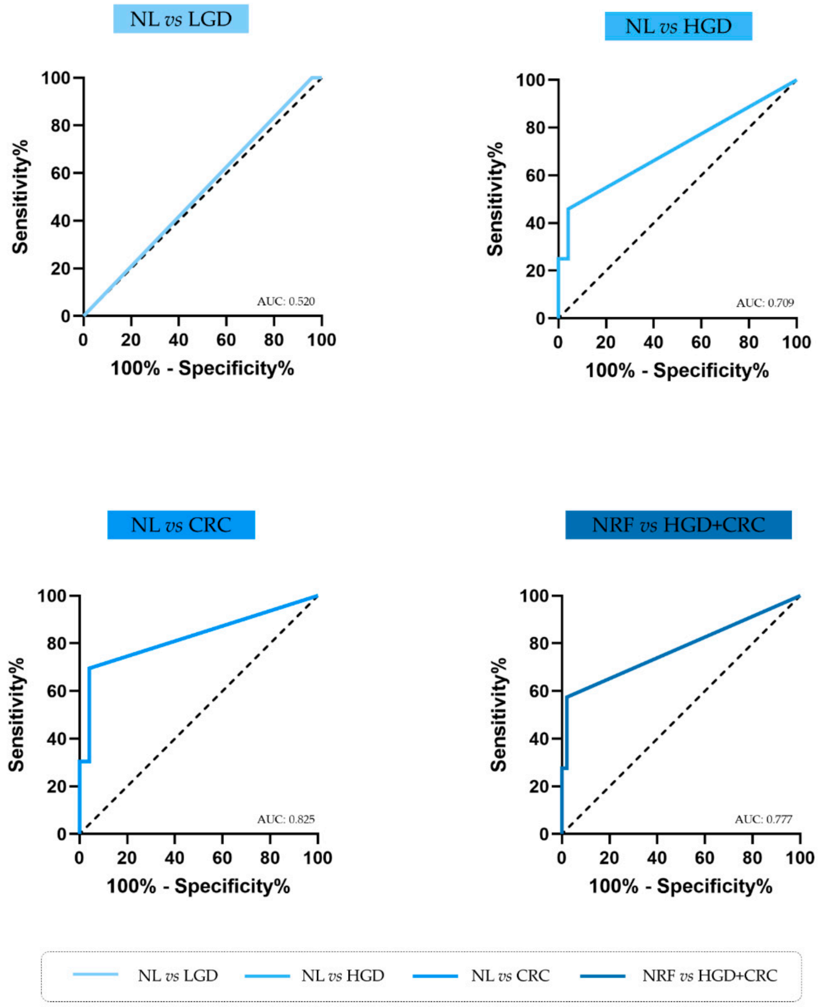

Figure S1: ROC analysis and AUC values of stool STn. Abbreviations: NL – No Lesion; LGD – Low-Grade Dysplasia; HGD – High-Grade Dysplasia; CRC – Colorectal Cancer; NRF – Non-Relevant Findings (NL + LGD).

Table S1: Correlation between STn expression and age, sex, localization, number of polyps, size of polyps, TNM staging and clinical stage. Bold values indicate p-value < 0.05.

		STn		
		Negative n(%)	Positive n(%)	p-value
**Age**	< 62	38 (57)	12 (43)	0.217
	≥ 62	29 (43)	16 (57)	
**Sex**	Female	32 (48)	13 (43)	0.906
	Male	35 (52)	15 (57)	
**Localization**	Right colon	14 (32)	6 (22)	0.071
	Left colon	25 (57)	11 (41)	
	Rectum	5 (11)	10 (37)	
**Number of polyps**	<2	25 (68)	3 (27)	**0.017**
	≥3	12 (32)	8 (73)	
**Size of polyps**	<1mm	19 (51)	1 (9)	**0.013**
	≥1mm	18 (49)	10 (91)	
**Tumour (T)**	T1	2 (29)	5 (31)	0.697
	T2	2 (29)	2 (12)	
	T3	3 (42)	7 (44)	
	T4	-	2 (13)	
**Lymph node metastasis (N)**	N0	6 (86)	13 (81)	0.805
	N1	0	1 (7)	
	N2	1 (14)	2 (12)	
	N3	0	0	
**Clinical Stage**	I	4 (57)	6 (38)	0.670
	II	2 (29)	6 (38)	
	III	1 (14)	4 (24)	
	IV	0	0	

Table S2: Performance characteristics of stool STn in the identification of individuals from different clinical groups.

Clinical Groups	AUC (95% CI)	Youden Index	
		Sens (%) (95% CI)	Spec (%) (95% CI)
**LGD [a]**	0.520 (0.356–0.686)	100 (86–100)	4 (1–20)
**HGD [a]**	0.709 (0.560–0.859)	46 (28–65)	96 (80–100)
**CRC [a]**	0.825 (0.698–0.952)	70 (49–84)	96 (80–100)
**HGD + CRC [b]**	0.777 (0.679–0.874)	58 (43–70)	98 (89–100)

Abbreviations: LGD – Low-Grade Dysplasia; HGD – High-Grade Dysplasia; CRC – Colorectal Cancer; HGD + CRC – Advanced Lesions; NR – Non-Relevant Lesions; AUC - Area under Curve; Sens - Sensitivity; Spec - Specificity; CI – Confidence Intervals. [a] Negative Category: individuals without lesions detected in colonoscopy; [b] Negative Category: individuals with non-relevant findings in colonoscopy.
